# Back to the future; an exploration of cardiac mass measurements

**DOI:** 10.1186/1532-429X-16-S1-T2

**Published:** 2014-01-16

**Authors:** Geetha Rayarao, Mark Doyle, Diane V Thompson, Sahadev T Reddy, Ronald B Williams, June A Yamrozik, Robert W Biederman

**Affiliations:** 1 Cardiac MRI, Allegheny General Hospital, Pittsburgh, Pennsylvania, USA

## Background

Historically, many of the original approaches to identify blood pool and myocardium relied on 'region growing' techniques, which seek out boundaries based on a combination of grey level intensities and edge sharpness. However, these approaches have been largely abandoned due to the poor contrast quality of the then standard, gradient echo imaging. In the modern era of superior image quality with SSFP imaging, revisiting the region growing approach warrants serious consideration, since it potentially leads to a more accurate assessment cardiac chamber volume and tissue mass. Hypothesis: Measurements of cardiac mass by ATMT operating on SSFP images is highly correlated with explanted weights.

## Methods

Explanted hearts from heart transplant recipients (n = 45) were prepared and weighed using a high-fidelity scale. CMR imaging was performed using SSFP-oriented in the short axis, measured contiguously from base to apex. Using a readily available Automatic Thresholding program, segmentation of the slices was achieved in combination with Manual Trimming (ATMT) of the extraneous signal in a 3D model by an independent and blinded reader. Thresholding employed upper and lower limits of intensities to select the myocardium, and trimming on the 3D model allowed intuitive interaction. The accuracy of this model was demonstrated by comparing the cardiac mass measurements by ATMT to that obtained from pathology, analyzed using correlation and Bland-Altman methods.

## Results

**Figure 1:**
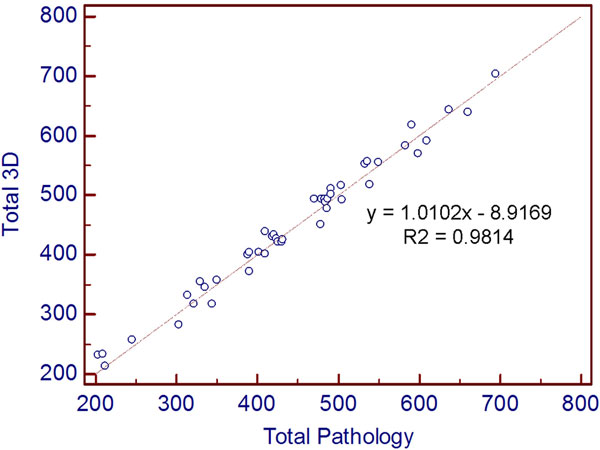
**Correlation plot**.

**Figure 2:**
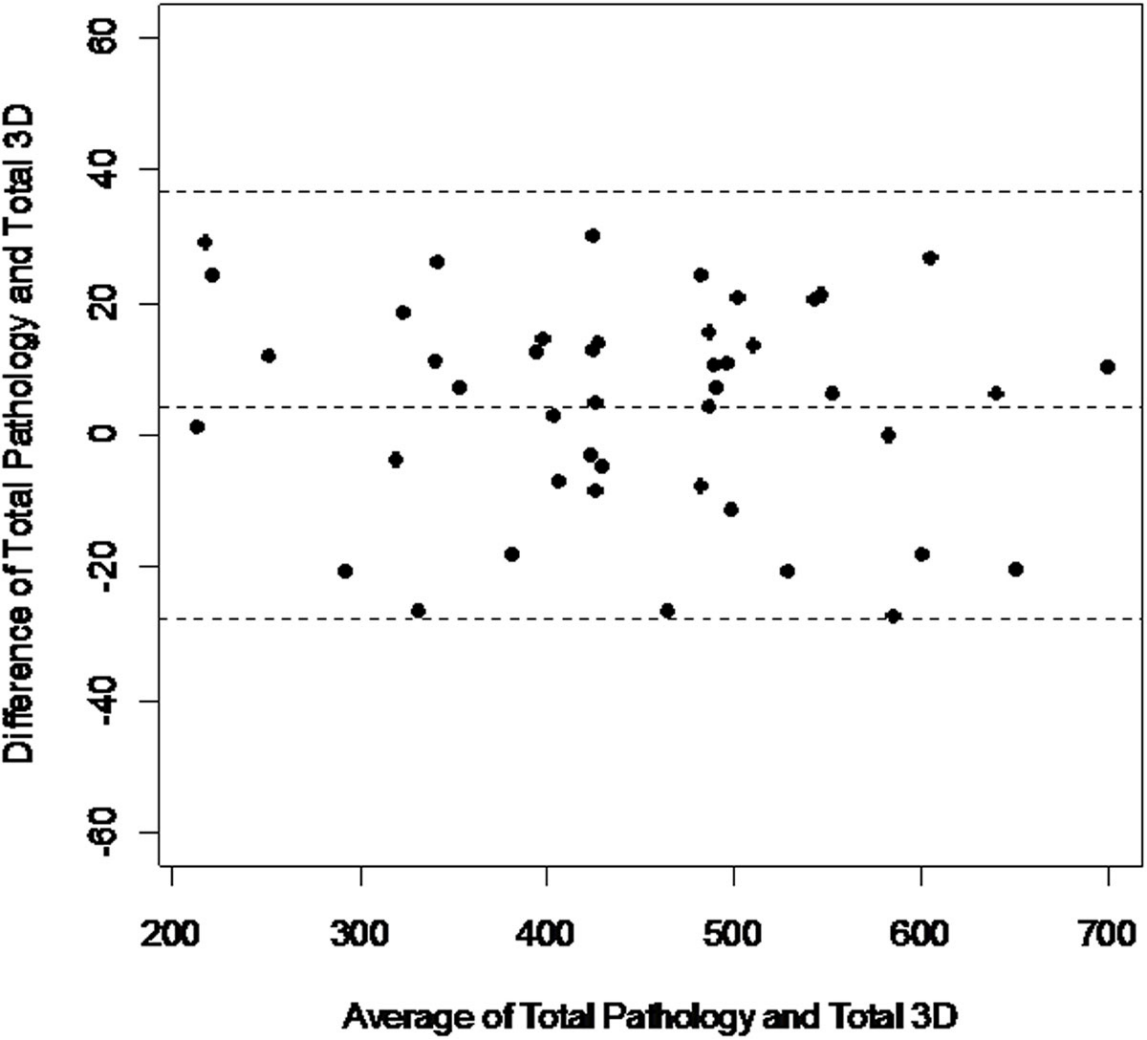
**Bland-Altman plot**.

Total cardiac mass measured from ATMT strongly correlated (Figure 1) with the pathology weight (y = 1.0102x - 8.9169; R = 0.991, p < 0.01). The Bland-Altman analysis (Figure 2) shows a standard deviation of 16.1 with a positive offset of 4.4 gms. A sub-analysis of 12 hearts with RVAD/LVADs removed showed a higher standard deviation (19) compared to the entire data set (16.1). Typically, hearts with no implants gave more accurate results.

## Conclusions

The correlation slope of 1.01 demonstrates excellent linearity over a large range: 500 gms (200 - 700 gms). Importantly, Bland-Altman analysis showed that the errors were distributed uniformly, which is consistent with over/under estimation due to partial volume error, more dominant in 2D vs. 3D imaging. Indicating that in-vivo imaging with high resolution and true 3D acquisition holds great promise. Hearts with RVAD/LVAD removal disruption showed higher errors. In future in-vivo work, this might have a significant impact in calculation of EF since the contouring methods might systematically over estimate the chamber volume at end systole. This indicates that 'thresholding', which is a basic region-growing technique, could be used to better identify and define cardiac tissue as we strive for more accuracy. Thus, in conclusion, the conventional approach of excluding 'papillary/trabecular mass' should be reconsidered. If validated, a wholesale change to the current contouring approaches would be indicated, with implications for our SCMR Society.

## Funding

Internal.

